# Primary Intraosseous Synovial Sarcoma with Molecular Confirmation: Expanding and Clarifying the Spectrum of This Rare Neoplasm

**DOI:** 10.1155/2020/5492754

**Published:** 2020-01-28

**Authors:** Kelsey E. McHugh, John D. Reith, Nathan W. Mesko, Scott E. Kilpatrick

**Affiliations:** ^1^Department of Anatomic Pathology, Cleveland Clinic, 9500 Euclid Avenue, Cleveland, OH 44195, USA; ^2^Department of Orthopedic Surgery, Cleveland Clinic, Cleveland, OH 44195, USA

## Abstract

Synovial sarcoma is a well-known malignant tumor usually originating within deep soft tissues of the lower extremities of adolescents and young adults. Rare radiologically confirmed examples of primary bone synovial sarcoma have been documented, generally in isolated case reports. Herein, we report two cases of primary intraosseous synovial sarcoma, with molecular confirmation, involving the left humerus of a 45-year-old female and the right fourth metatarsal bone in a 36-year-old male. Additionally, we clarify the spectrum of primary intraosseous synovial sarcoma by separately analyzing reported cases with radiographic confirmation of bone origin and molecular support for the diagnosis. There are clinicopathologic differences between those tumors with documented molecular confirmation and those lacking such confirmation, specifically regarding their anatomic distribution (*p* < 0.0001). Regarding the radiology of our two cases, the humeral lesion appeared almost entirely intramedullary without soft tissue extension; the midfoot lesion demonstrated a destructive, metatarsal-centered bone lesion, initially thought clinically to represent primary bone osteosarcoma. The diagnoses of monophasic synovial sarcoma were rendered via core needle biopsies, with molecular FISH confirmation of *SYT* gene rearrangement. Clinical follow-up data was only available for the female patient with the primary humeral lesion, who underwent surgical resection, with no local recurrence or distant metastasis at 7 months postsurgery. To our knowledge, these are the first reported examples of molecularly confirmed, primary intraosseous synovial sarcomas of the humerus and metatarsal bones. Primary intraosseous synovial sarcomas with molecular confirmation differ clinically from those lacking it; however, the demographic features and metastatic potential appear similar to primary soft tissue synovial sarcoma.

## 1. Introduction

Synovial sarcoma is a malignant mesenchymal neoplasm most commonly arising in the deep soft tissues of the extremities in young to middle-aged adults. It accounts for approximately 5-10% of soft tissue sarcomas, often arising near joint spaces, and very rarely presents primary to the viscera or bone [1]. A specific disease-defining chromosomal translocation, involving the *SYT* gene on chromosome 18 and either the *SSX1* or *SSX2* gene on chromosome X, is identified in the overwhelming majority (over 90%) of synovial sarcomas [1]. Examination of the peer-reviewed English-language literature reveals a total of 11 reported cases of primary intraosseous synovial sarcoma (8, monophasic; 3, biphasic), when excluding the two cases described herein, with radiologic support and molecular and/or cytogenetic confirmation of the diagnosis (Table 1). The appendicular skeleton, especially the long bones of the lower extremities, is far more commonly involved than the axial skeleton. Specific primary sites of involvement have included the tibia (5 cases) [2–5], ulna (2) [6, 7], radius (1) [8], fibula (1) [9], femur (1) [10], and thoracic spine (1) [11]. An additional 26 reported cases, lacking molecular or cytogenetic confirmation of the diagnosis, are also identifiable within the English-language literature (Table 2), some of which may not represent “true” synovial sarcomas [12–22]. Among these cases, there appears to be a clear predominance of head and neck origin, especially the osseous mandible, an anatomic site which has never had a molecularly confirmed and clearly documented example. Herein, we report two cases of primary intraosseous synovial sarcoma, primary to the humerus and metatarsal bones, with molecular confirmation. Our paper also includes a comprehensive literature review of this rare entity, with emphasis (and distinction) of those cases with adequate radiographic, molecular, and/or cytogenetic confirmation.

## 2. Materials and Methods

This study was approved by the Cleveland Clinic Institutional Review Board (IRB#19-680). The in-house and consultative files of 2 coauthors (J.R. and S.K.) were reviewed for diagnoses of primary intraosseous synovial sarcoma. Routine hematoxylin and eosin stained slides were rereviewed to confirm diagnoses. For inclusions, tumors had to demonstrate a clearly unequivocal intraosseous epicenter and had to have undergone molecular confirmation of the rendered histologic diagnosis. Clinical and radiographic features were also rereviewed and compared. Treatment and clinical follow-up data were obtained from the patient files, when possible. Extensive review of the English language literature within the PubMed database, without restriction based on date of publication, was performed to identify previously reported cases of intraosseous synovial sarcoma with and without molecular confirmation of the diagnosis. Fisher's exact test was performed to assess for statistical significance between molecularly confirmed and non-molecularly confirmed cases of intraosseous synovial sarcoma regarding anatomic site distribution. Statistical analysis was performed using online statistical software (GraphPad; La Jolla, CA).

## 3. Case Presentations

Two cases were identified. Case presentation details are below.

### 3.1. Case 1

#### 3.1.1. Clinical Findings

**Figure 1:**
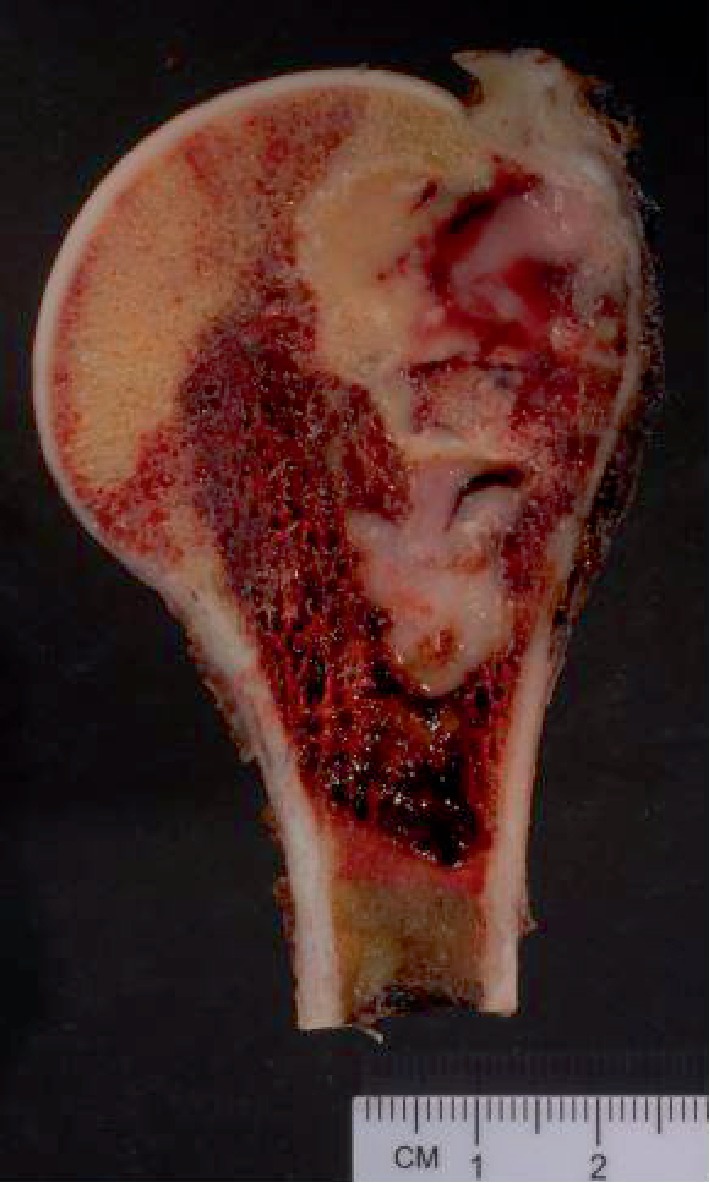
Case 1: proximal left humerus resection showing a fleshy tan-gray lesion involving the intramedullary space with destruction of overlying cortical bone and associated foci of hemorrhage and necrosis.

**Figure 2:**
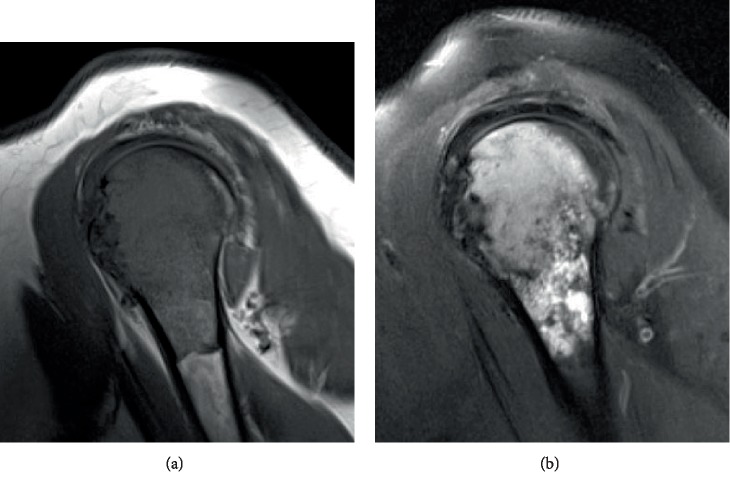
Case 1: sagittal left shoulder MRI showing a 6 cm intramedullary lesion involving the proximal left humerus with associated periostitis. The lesion was hypointense on T1-weighted images (a) and hyperintense on T2-weighted images (b).

A 45-year-old female presented with a 2-year history of intermittent and worsening left shoulder pain, which recently began radiating to her left fingers and was associated with numbness and restricted range of motion. She reported physical aggravation to the area 3 days prior to presentation, secondary to moving luggage. There was no prior history of malignancy. Physical examination demonstrated limited active range of motion secondary to pain as well as tenderness to palpation over the left bicipital groove. No external abnormalities or palpable masses were noted. Magnetic resonance imaging (MRI) revealed a marrow-replacing lesion involving the proximal 6 cm of her left humerus (epiphyseal and metaphyseal), with associated periostitis, but minimal cortical destruction and no definite soft tissue extension (Figure 1). It was hypointense on T1-weighted images and hyperintense on T2-weighted images (Figure 2). Whole body bone scan showed increased lesional scintigraphic uptake. No additional osseous lesions were identified. Computed tomography (CT) of the chest, abdomen, and pelvis is negative for lymphadenopathy or metastasis. Fluoroscopy-guided core needle biopsy was performed using a 15-gauge Lee-Lok needle. Five cores were obtained and the diagnosis of synovial sarcoma was confirmed (see below pathologic findings). Subsequently, the patient underwent radical resection of the proximal 7.5 cm of the left humerus with humeral reconstruction and reverse total shoulder prosthesis. Approximately one month postoperation, the patient began systemic adjuvant chemotherapy with doxorubicin and ifosfamide. She completed six cycles. Approximately seven months postoperation, PET scan confirms that she remains free of disease recurrence or metastasis.

#### 3.1.2. Histologic Findings

**Figure 3:**
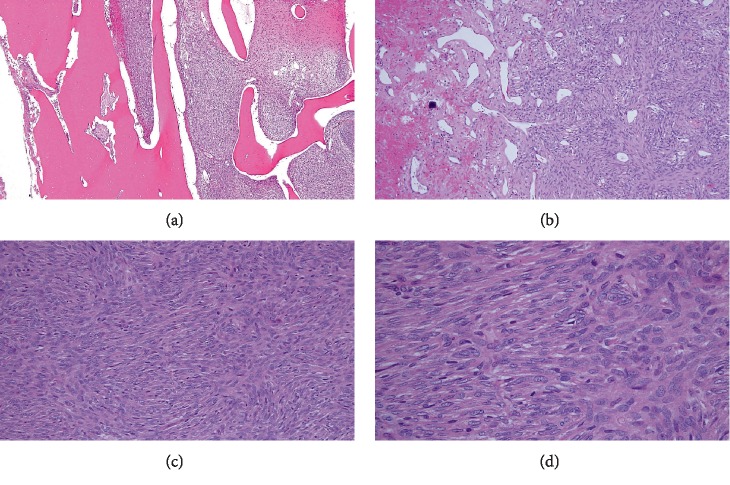
Case 1: resection specimen demonstrating cortical erosion with bony permeation (a). Scattered foci of hemangiopericytoma-like vasculature were present (b). The tumor was monophasic, comprised of bland, ovoid spindle cells arranged in storiform (c) and fascicular (d) patterns.

**Figure 4:**
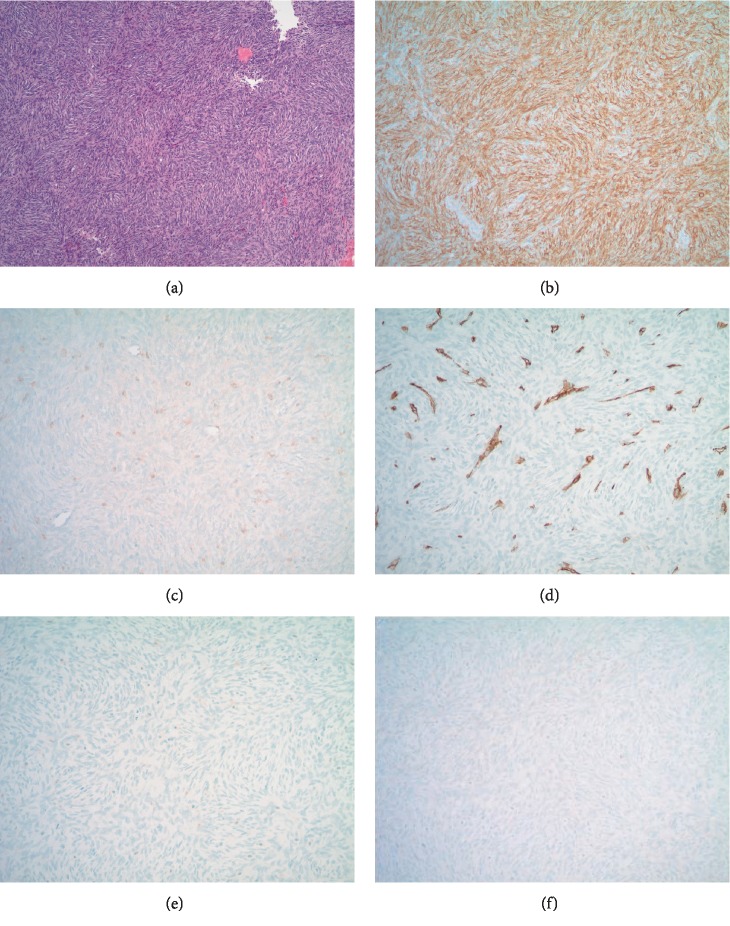
Case 1: H&E-stained core needle biopsy material (a) and associated immunohistochemistry results demonstrating diffuse membranous positivity for CD99 (b) and focal positivity for EMA (c). CD34 highlights the vasculature of the tumor and is negative in tumor cells (d). The tumor cells are also negative for pankeratin AE1/3 (e) and STAT6 (f).

**Figure 5:**
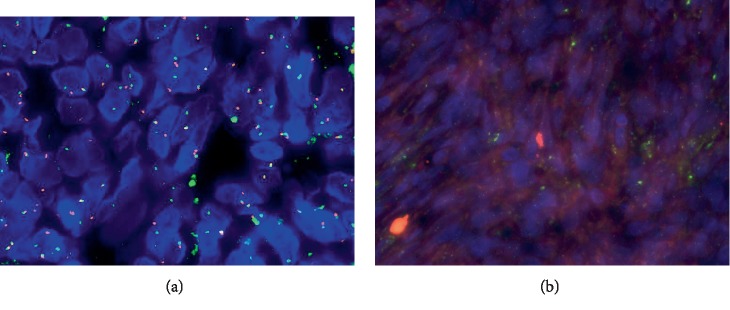
Cases 1 and 2: positive fluorescence in situ hybridization (FISH) analysis for *SYT* (*SS18*) gene rearrangement, performed at the Cleveland Clinic, demonstrated by an abnormal signal pattern seen as disruption of the *SYT* gene through the breaking apart of the red and green probe signals. In case 2, *SYT* gene rearrangement was seen in 94% of tumor nuclei (a). In case 1, *SYT* gene rearrangement was seen in 76% of tumor nuclei (b).

Core needle biopsy and resection material was embedded in paraffin, sectioned, and stained with hematoxylin and eosin. Histologic sections revealed a densely cellular spindle cell neoplasm arranged in storiform patterns as well as short fascicles, eroding the cortex and permeating native bony trabeculae. A background hemangiopericytoma-like vascular proliferation was prominent and, focally, a prominent collagenous component interlaid between the neoplastic cells. Areas of necrosis were also noticeable (Figure 3), as was scattered dystrophic calcification. Immunohistochemically, the neoplastic cells demonstrated diffuse immunohistochemical reactivity for CD99, were focally positive for EMA, and were negative for STAT6, AE1/3, and CD34 (Figure 4). FISH analysis for the *SYT* (*SS18*) gene rearrangement, performed at the Cleveland Clinic on biopsy material that did not undergo decalcification, was positively confirmed (Figure 5). These findings are consistent with a monophasic (fibrous) primary intraosseous synovial sarcoma.

### 3.2. Case 2

#### 3.2.1. Clinical Findings

**Figure 6:**
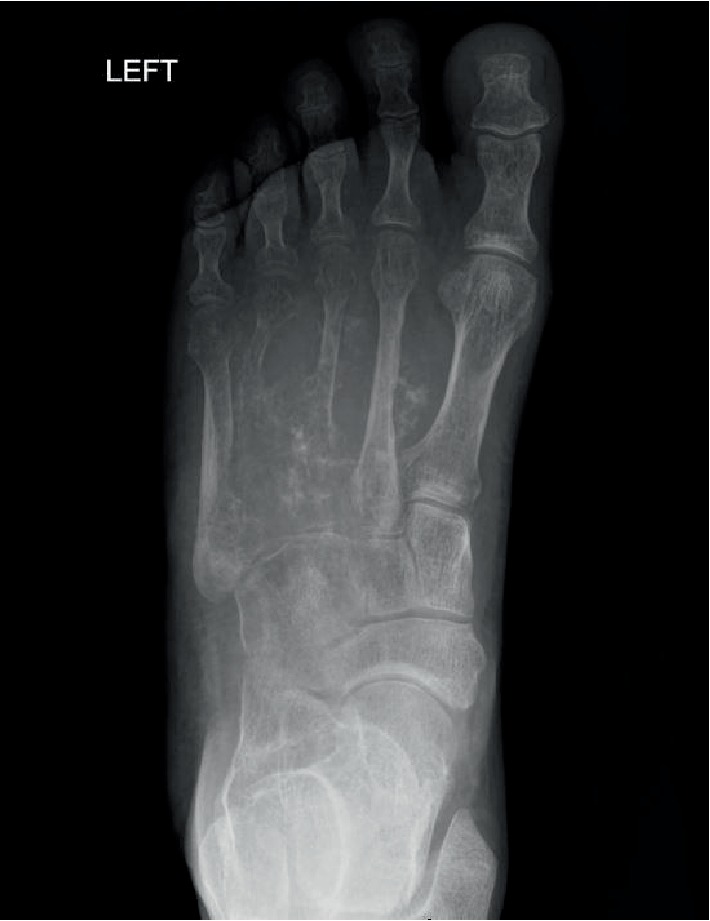
Case 2: anteroposterior left foot radiograph showing a lytic and destructive mass centered on the 4th metatarsal, with destructive extension to the 3rd metatarsal and adjacent cuneiform bones. Punctate calcifications consistent with foci of mineralization can be seen throughout the lesion.

A 36-year-old male with a history of diabetes mellitus presented with a 1.5-year history of intermittent and gradually worsening left lateral foot pain, primarily involving the heel and arch. There was no prior history of trauma or malignancy. Plain film radiographs revealed a lytic and destructive mass involving and centered on the left midfoot, associated with virtually complete destruction of the 4th metatarsal, with extension to the 3rd metatarsal and adjacent cuneiform bones. There also were foci of mineralization (Figure 6). Prior to biopsy, he was evaluated at a major medical center, and the presumed clinical diagnosis was “osteosarcoma.” Core needle biopsy was performed at an outside institution and subsequently sent to the Cleveland Clinic for review, where a diagnosis of synovial sarcoma was confirmed. No additional clinical follow-up data is available.

#### 3.2.2. Histologic Findings

**Figure 7:**
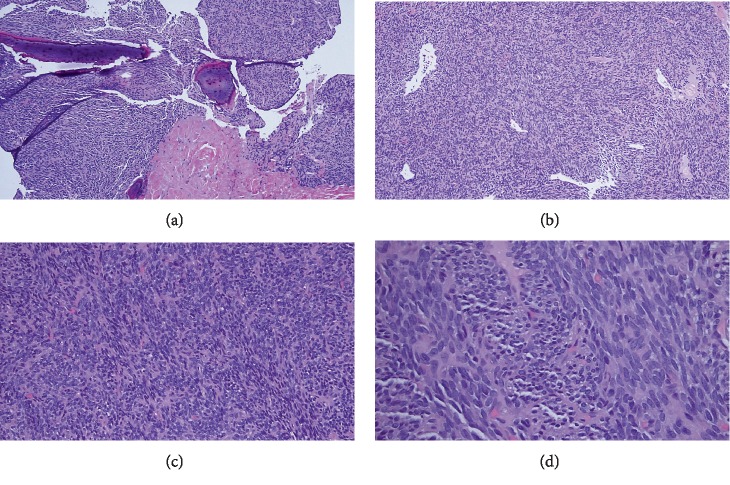
Case 2: core needle biopsy with permeation of spindle cells around bony trabeculae (a). Hemangiopericytoma-like vasculature was identifiable (b). The tumor was monophasic, comprised of uniform, ovoid spindle cells with scanty cytoplasm arranged in short fascicles (c, d).

**Figure 8:**
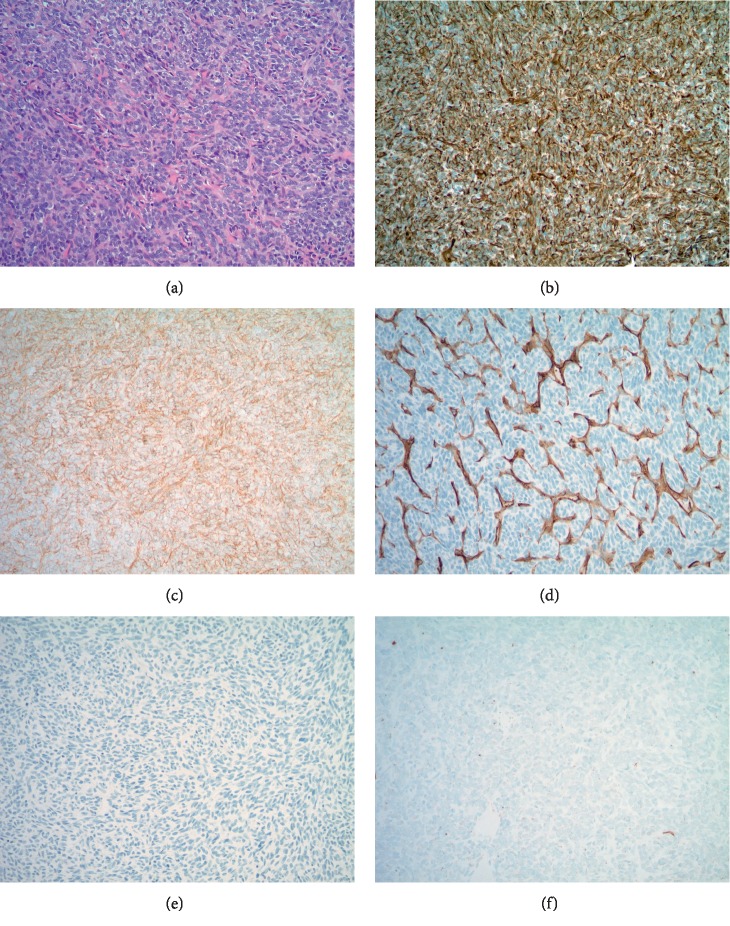
Case 2: H&E-stained core needle biopsy material (a) and associated immunohistochemistry results demonstrating strong diffuse cytoplasmic positivity for vimentin (b) and membranous positivity for CD99 (c). CD34 highlights the vasculature of the tumor and is negative in tumor cells (d). The tumor cells are also negative for pankeratin (e) and STAT6 (f).

Core needle biopsy material was embedded in paraffin, sectioned, and stained with hematoxylin and eosin. The specimen did not require decalcification. Histologic sections revealed a densely cellular spindled cell neoplasm largely arranged in short fascicles and sheets, permeating native bone trabeculae (Figure 7). A background hemangiopericytoma-like (i.e., solitary fibrous tumor-like, SFT-like) vascular proliferation was obvious. Individual cells appeared tightly packed with variable amounts of collagen in the background stroma. Immunohistochemically, the neoplastic cells demonstrated diffuse membranous immunoreactivity for CD99, while STAT6, CD34, CK20, SMA, desmin, pankeratin, CAM5.2, Melan A, and S100 were negative (Figure 8). FISH analysis for the *SYT* (*SS18*) gene rearrangement, performed at the Cleveland Clinic, was positively confirmed (Figure 5). These findings are consistent with a monophasic (fibrous) primary intraosseous synovial sarcoma.

## 4. Discussion

Synovial sarcoma was first described in the late 1800s [23] and first acquired its moniker, by which we still refer to it today, in 1914 at the behest of Jones and Whitman [24]. The term “synovial sarcoma” is a misnomer, as the tumor virtually never arises from within a joint (intra-articular) and lacks both ultrastructural and immunohistochemical evidence linking it to the normal (or even reactive) synovium. Adding to this controversy, synovial sarcoma has been documented as arising in a variety of anatomic sites, including but not limited to the lung and pleura [25–29], heart and pericardium [30, 31], kidney [12, 32–37], prostate [38], and gastrointestinal tract [39–43]. Regarding connective tissues, synovial sarcoma may have its origins within intramuscular [44], para-articular [23], intraneural [45–47], or intraosseous tissues [2–11]. For the above reasons, some have proposed renaming synovial sarcoma as “carcinosarcoma of connective tissue,” reflecting the concept of true epithelial differentiation in association with a spindle cell sarcoma and the absence of true synovial differentiation [48, 49]. Nevertheless, despite the complications of its nomenclature and the fact that its histogenesis remains unclear, synovial sarcoma is a recognized and well-defined clinicopathologic entity, which harbors a reproducible cytogenetic abnormality, t(X;18)(p11.2;q11.2), associated with the *SYT/SSX* fusion.

True primary intraosseous synovial sarcoma, originating within the bone and associated with significant intramedullary and cortical bone destruction, remains a very rare entity, largely limited to isolated case reports. For the purposes of reviewing the literature in regard to demographics, pathologic features, and clinical course, we have provided two tables: Table 1 summarizes reported primary intraosseous synovial sarcomas with documented molecular and/or cytogenetic confirmation of the diagnosis, while Table 2 summarizes those reported cases lacking this molecular/cytogenetic confirmation. In some instances, it was difficult to determine which reported cases were truly intraosseous in origin versus those that secondarily involved the bone (e.g., soft tissue origin). In our review of the literature, we included only those cases in which detailed radiographic descriptions and/or images confirmed primary bone origin.

When comparing the demographic data of molecularly/cytogenetically confirmed primary intraosseous synovial sarcomas with reported cases lacking this confirmation, it is interesting to note some differences in mean patient age, sex ratios, and anatomic distributions (*p* < 0.0001). Reported cases with molecular/cytogenetic confirmation had an average patient age of 43 years (range, 21 to 77) and a male : female ratio of 2.25, while those cases lacking molecular/cytogenetic confirmation had an average patient age of 34.8 years (range, 14 to 86) and a male : female ratio of 0.6. Those with molecularly/cytogenetically confirmed primary intraosseous synovial sarcoma had the disease nearly exclusively limited to the long bones of the appendicular skeleton (excluding 1 case involving a thoracic vertebra), most commonly the lower extremities, with the tibia representing the single most common anatomic site. Cases lacking molecular/cytogenetic confirmation predominantly involved the head and neck, specifically the mandible and maxilla (18/35 cases, 51.4%). To our knowledge, not a single case of primary intraosseous synovial sarcoma of the head and neck has ever been confirmed via molecular or cytogenetic means.

It is well established that primary soft tissue synovial sarcoma may secondarily involve the underlying bone in up to 20% of cases [23, 50]. In these instances, the degree of bone involvement almost always is minimal, and the epicenter of the lesion remains in soft tissue. Among cases with adequate radiologic descriptions and images, primary bone synovial sarcomas are typically lytic and destructive lesions, with ill-defined margins, and almost always associated with soft tissue extension [3–6, 8, 51]. In the long bones, the lesions may be epiphyseal, metaphyseal, or diaphyseal, leading to a wide range of prebiopsy differential diagnoses. Rarely, primary bone synovial sarcoma may appear deceptively benign [7]. By MRI scan, intraosseous synovial sarcomas are typically isointense to hypointense on T1-weighted images and hyperintense on T2-weighted images [6–8, 11]. In both of our cases, the tumors were primarily lytic lesions centered in and primarily localized to the bone, with ill-defined margins. For case 1 (humeral lesion), there was minimal soft tissue extension, only really appreciated upon evaluation of the gross specimen. Regarding case 2 (foot lesion), the 4th metatarsal was virtually entirely destroyed, with extension into the 3rd metatarsal and adjacent cuneiforms. Indeed, case 2 was initially thought to represent a primary bone osteosarcoma, clinically and radiologically, prior to core needle biopsy and evaluation at the Cleveland Clinic. As a general rule, with the exception of some flat bones (e.g., the scapula), significant bone destruction, even with soft tissue extension, virtually always equates with primary origin within the bone.

Regarding the clinical course of patients with molecularly/cytogenetically confirmed primary intraosseous synovial sarcoma, the literature in combination with this report reveals a near-even split between those patients who experience local recurrence and/or metastasis and those whose clinical course lacked evidence of recurrent or progressive disease post-initial surgical intervention. Eleven of 13 patients (84.6%) had relevant reported clinical management data regarding their initial presentation, and 9 (69.2%) had reported clinical follow-up data. Of 11 patients, 6 (54.5%) reportedly received adjuvant or neoadjuvant chemotherapy [3, 4, 8, 10, 11]. In 5 of 6 cases, the chemotherapy regimen was disclosed [4, 8, 10, 11]. Four of 5 patients received ifosfamide and/or doxorubicin as part of their chemotherapy regimen: 3 patients received both chemotherapeutic agents (1 additionally received dacarbazine) and 1 patient received doxorubicin with olaratumab [4, 10, 11]. A single patient received cis-platinum and pirarubicin [8]. Ultimately, 2 patients experienced local recurrence (both in the tibia), 4 patients had distant metastases (3 pulmonary and 1 inguinal lymph node), and 5 patients did not experience recurrent or metastatic disease at initial presentation or during their subsequent clinical course (follow-up, 2 to 24 months; average follow-up, 12.5 months). In all, 1 of the 9 patients (10%) for which follow-up clinical data is reported was dead of disease (DOD). The remaining 8 patients were alive without evidence of further progression of aforementioned local recurrences or metastases at 2 to 96 months follow-up (average follow-up, 33.5 months). For those cases that lacked molecular confirmation of diagnosis, 20 of 26 patients (76.9%) had reported clinical follow-up data. Ultimately, 6 of 20 patients (30%) were DOD. The clinical course of this cohort detailed 9 patients (45%) that experienced local recurrence and/or metastasis (6 local recurrences and 5 metastases), whereas 11 patients (55%) were without subsequent incident (follow-up, 2 to 90 months).

At least some examples of what was originally thought to represent primary bone hemangiopericytoma (i.e., solitary fibrous tumor, SFT) now appear to be misdiagnosed synovial sarcomas [9]. Both of our cases of monophasic spindle cell synovial sarcoma demonstrated a prominent hemangiopericytoma- (SFT-) like vascular pattern and were negative, immunohistochemically, for cytokeratins. Interestingly, the majority (10/13 cases, 76.9%) of molecularly confirmed primary intraosseous synovial sarcomas are histologically monophasic (see Table 1). Regarding the potential for a biphasic histologic appearance, it has recently been documented that primary bone synovial sarcoma, especially given the fact that it commonly involves the tibia, may be misdiagnosed as adamantinoma [5]. The possibility of misdiagnoses of other potentially biphasic-appearing malignancies that arise in (or may involve) jaw bones, such as ameloblastoma, including “malignant” ameloblastoma/ameloblastic carcinoma, in younger patients, or sarcomatoid carcinoma, in older patients, should certainly be considered in the differential diagnoses of “synovial sarcoma” from the craniofacial region and may account for discrepancies in the literature. Because of the rarity of the disease and the difficulty of establishing the diagnosis of primary bone synovial sarcoma, we believe that many of the previously reported examples of “synovial sarcoma” lacking molecular confirmation, especially those of the head and neck, may not represent synovial sarcomas.

## 5. Conclusions

In summary, primary intraosseous synovial sarcoma, with supportive radiologic and molecular and/or cytogenetic confirmation, is an extraordinarily rare entity, most commonly arising in the appendicular skeleton, especially the long bones of the lower extremities (more specifically, the tibia). The vast majority are lytic, destructive lesions, usually associated with soft tissue extension. Histologically, most are monophasic spindled cell (fibrous). To our knowledge, including our cases, there are only 13 molecularly confirmed examples in the literature. We have described the first cases, also with molecular confirmation, of primary humeral and primary metatarsal intraosseous synovial sarcoma. It appears that cases of primary intraosseous synovial sarcoma with molecular or cytogenetic confirmation of the diagnosis, though limited in number, have demographic features and metastatic potential similar to primary soft tissue synovial sarcoma, demonstrating a male predominance and a rate of metastasis nearing 40%. Regarding overall prognosis, the paucity of bona fide cases combined with the limited available clinical follow-up data precludes definitive conclusions. Reported examples of intraosseous synovial sarcoma without molecular confirmation may represent a diverse group of neoplasms, many of which may not be “true” synovial sarcoma, which is highlighted by the significant difference (*p* < 0.0001) in anatomic site distribution between those with and those lacking molecular confirmation of diagnosis. Given the rarity of the disease, establishing an accurate diagnosis of intraosseous synovial sarcoma necessitates radiographic correlation coupled with judicious use of ancillary studies especially molecular testing, such as FISH analysis, to ensure an accurate diagnosis.

## Figures and Tables

**Table 1:** Reported cases of primary intraosseous synovial sarcoma with molecular confirmation of the diagnosis.

Author	Year	No. cases	Age	Sex	Anatomic site	Extraosseous component	Morphology	Molecular confirmation	Clinical management	Clinical follow-up
Cohen et al.	1997	1	22	M	Proximal tibia	Yes	Biphasic	FISH	NA	NA

Hiraga et al.	1999	1	67	M	Distal radius	No	Monophasic	RT-PCR	Neoadjuvant chemotherapy; wide resection	Pulmonary metastases 8 months postoperative; metastases in radius treated with XRT; NED 2.5 years

O'Donnell et al.	2006	1	37	M	Proximal ulna	Yes	Monophasic^∗^	RT-PCR	Amputation	NED 1 year

Jung et al.	2007	1	21	F	Distal tibia	Yes	Monophasic	RT-PCR	Neoadjuvant chemotherapy	NA

Verbeke et al.	2010	1	73	F	Fibula	No	Monophasic	FISH	Amputation	Pulmonary metastases after 0.25 years; ultimately DOD

Beck et al.	2011	1	53	M	Proximal tibia	Yes	Biphasic	FISH	Proximal tibial resection; adjuvant chemotherapy	Local recurrence at 3 years; AKA & chemotherapy; NED 8 months

Cao et al.	2014	1	26	M	Thoracic spine	Yes	Biphasic	RT-PCR	En bloc resection; adjuvant chemotherapy & XRT	NED 1 year

Fujibuchi et al.	2019	1	77	F	Distal ulna	No	Monophasic	RT-PCR	Wide resection	NED 2 years

Horvai et al.	2019	2	33	M	Tibia diaphysis	No	Monophasic	FISH	Curettage	Local recurrence & metastasis to inguinal lymph node; adjuvant chemotherapy; NED 7 years
			36	M	Tibia diaphysis	No	Monophasic	FISH	En bloc resection	Local recurrence; NED 8 years

Caracciolo et al.	2019	1	33	M	Proximal femur	Yes	Monophasic	RT-PCR	Pulmonary metastases at initial presentation; neoadjuvant chemotherapy; proximal femoral resection	Progressive pulmonary metastases; adjuvant chemotherapy; no additional follow-up provided

McHugh et al.	2019	2	45	F	Humerus	No	Monophasic	FISH	Proximal humeral resection; adjuvant chemotherapy	NED 2 months
			36	M	Metatarsal	Yes	Monophasic	FISH	NA	NA

Total cases: 13. ^∗^Poorly differentiated (the remainder of the monophasic tumors is of the spindle cell/fibrous type). Abbreviations: M: male; F: female; FISH: fluorescent in situ hybridization; RT-PCR: reverse-transcription polymerase chain reaction; NA: not applicable (not reported); XRT: radiation therapy; NED: no evidence of disease; DOD: dead of disease.

**Table 2:** Reported cases of primary intraosseous synovial sarcoma without molecular and/or cytogenetic confirmation of the diagnosis.

Author	Year	No. cases	Age	Sex	Anatomic site	Extraosseous component	Morphology	Clinical management	Clinical follow-up
Rose et al.	1982	1	16	F	Distal femur	No	Biphasic	Subtrochanteric amputation	NA
Nakajo et al.	2005	1	86	M	Sternum	Yes	Monophasic	NA	NA
Tilakaratne et al.	2006	1	29	F	Mandible ramus	No	Monophasic	Adjuvant XRT	NED 2 years
Tao et al.	2011	1	20	F	Posterior mandible	No	Monophasic	Extensive mandibulectomy; adjuvant XRT	NED 6 months
Khalili et al.	2012	1	76	M	Posterior mandible	No	Monophasic	Lymph node metastases at the time of diagnosis	DOD at 2 mo. postdiagnosis
Zulkarnaen et al.	2012	1	57	M	Proximal femur	Yes	Biphasic	Neoadjuvant chemotherapy; wide excision; adjuvant chemotherapy	Pulmonary metastasis; DOD
Kim et al.^∗^	2013	1	17	M	C3 vertebra	No	Monophasic	NA	NA
Bansal et al.	2015	1	16	F	Mandible	Yes	Monophasic	NA	NA
Liu et al.	2015	15	14-62y	5M, 10F	Maxilla (7) Mandible (8)	NA	6 biphasic, 9 monophasic	Lymph node metastases in 1 patient; adjuvant XRT in 9 patients; adjuvant chemotherapy in 6 patients	6 patients with local recurrence (LR); 1 patient with pulmonary metastasis; 4 patients with LR DOD
Cardosa et al.	2016	1	56	F	Scapula	Yes	Monophasic	Neoadjuvant chemotherapy; en bloc resection	Subsequent lymph node metastases; excision and adjuvant XRT
Deyong et al.	2016	2	NA	NA	NA	NA	NA	NA	NA

Total cases: 26. ^∗^FISH molecular testing was performed and was negative for an SYT gene rearrangement. Abbreviations: F: female; M: male; NA: not applicable (not reported); XRT: radiation therapy; NED: no evidence of disease; DOD: dead of disease.
